# Will availability of inhaled human insulin (Exubera^®^) improve management of type 2 diabetes? The design of the *Real World trial*

**DOI:** 10.1186/1745-6215-7-25

**Published:** 2006-08-10

**Authors:** Nick Freemantle, Thomas R Strack, the Real World Trialists

**Affiliations:** 1 University of Birmingham, Birmingham, UK; 2 Pfizer Inc, New York, NY, USA

## Abstract

**Background:**

Common deterrents to insulin therapy for both physicians and patients are the complexity and burden of daily injections. In January 2006, the first inhaled human insulin (INH, Exubera^® ^(insulinhuman [rDNA origin])InhalationPowder) was approved for use in adult patients with type 1 diabetes mellitus (T1DM) or type 2 diabetes mellitus (T2DM) in the United States and European Union. Results from the INH clinical trial program have shown comparable efficacy of INH to subcutaneous (SC) insulin and superior efficacy versus oral antidiabetic agents; thus providing effective glycemic control in adult patients with T2DM without the requirement for preprandial injections. However, because subjects in those trials were randomized to either INH or an alternative, the studies could not estimate the effect of INH on patient acceptance of insulin therapy. Therefore, traditional study designs cannot provide answers to important and practical questions regarding real world effectiveness, which is influenced by psychological and other access barriers.

**Methods:**

To overcome these limitations, the Real World Trial was designed to estimate the effect of the availability of INH as a treatment option for glycemic control. A total of approximately 700 patients from Canada, France, Germany, Italy, Spain, United Kingdom, and the United States with T2DM poorly controlled by oral agent therapy will be randomized to two different treatment settings. Patients and clinicians in both groups (A & B) may choose from all licensed therapies for diabetes including SC insulin delivered by pens; INH will be an additional treatment option only available in Group A. The Real World Trial (Protocol A2171018) has been registered with ClincalTrials.gov, registration id NCT00134147.

**Results:**

The primary outcome for the trial will be the difference in mean glycosylated hemoglobin (HbA_1c_) at 6 months between groups. The design was based on a preceding feasibility study examining the theoretical effects of inhaled insulin availability on treatment choice in 779 patients. In that study, patients were three times more likely to choose insulin therapy when inhaled insulin was available.

**Conclusion:**

Innovations in study designs may provide an opportunity to reveal unbiased answers to important treatment questions that are more relevant to prescribers, funding agencies, and healthcare policymakers.

## 1.0 Background

Diabetes mellitus constitutes a major healthcare problem globally. In the United States, 10.3 million people were reported with this disease in 1997; and an additional 5.4 million people were suspected to have diabetes, but were not diagnosed nor treated [1]. The total number of individuals with identified diabetes is expected to increase to approximately 16 million by 2010 [2], and 22 million by 2025 [3]. Most patients have type 2 diabetes mellitus (T2DM), characterized by a combination of insulin resistance and progressive loss of insulin secretion. In approximately 58% of patients with T2DM, the disease is not sufficiently controlled based on American Diabetes Association (ADA) recommendations [4]. As a result, patients experience substantial morbidity and mortality due to complications resulting from chronic exposure to hyperglycemia [5-9] although major clinical trials have demonstrated that improved glycemic control can significantly reduce the risk of complications [10,11].

Oral antidiabetic agents (OAs) are the current mainstay of T2DM management, but their long-term efficacy is limited by the progressive and irreversible loss of β-cell function, whereas insulin therapy remains continually effective. However, initiation of insulin injections frequently is restricted as a "last resort" for a variety of reasons such as fear of needles, inconvenience, and technique. Indeed, several studies have shown delays of 4 years or longer in the uptake of insulin therapy by patients uncontrolled with multiple OAs [12,13]. This delay may accelerate β-cell failure and disease progression and hasten development of complications, highlighting the need for improved patient acceptance of insulin therapy so it may be used in a timelier and appropriate manner.

Non-invasive methods of insulin delivery, such as inhaled insulins, may increase the acceptance, and thus earlier initiation, of insulin therapy. The first inhaled human insulin (INH, Exubera^® ^(insulinhuman [rDNA origin]) Inhalation Powder) was recently approved by the European Commission and the US Food and Drug Administration to deliver effective glycemic control in adult patients with type 1 or type 2 diabetes. Phase 3 trials have demonstrated that INH is as efficacious as subcutaneously (SC) injected regular human insulin in type 1 diabetes (T1DM) [14,15] or T2DM [16], and superior to OAs in T2DM [17-20]. INH offers a number of potential advantages [14-29], including a time-action profile that is more suited for mealtime insulin administration compared with SC regular human insulin [21,22]. Another advantage of INH is that patients with T2DM who are poorly controlled with OAs are able to attain good glycemic control by simply adding INH to their oral regimen [18,19]. In a 12-week study by Rosenstock and colleagues, adjusted treatment group differences from baseline (9.5–9.6%) in glycosylated hemoglobin (HbA_1c_) of -1.2% and -1.7% were observed when INH was substituted for combination OA therapy or added to combination therapy, respectively [18]. Furthermore, Phase 3 trial results indicate that INH lowers HbA_1c _to ADA target levels (HbA_1c _< 7%) in more than one third of patients after only 3 months of treatment [16,18,20].

Evidence from completed Phase 2 and 3 studies up to 4 years in duration support that insulin administration via the lung is generally well tolerated in patients with T1DM or T2DM [12-20,23,24]. A slight, but clinically insignificant, decline in lung function was observed to a greater extent in patients treated with INH compared with control patients; this decline was nonprogressive and reversed after discontinuation of INH therapy [14-16,19,23,24]. The only significant clinical adverse effect associated with INH was cough, which was generally characterized as mild to moderate in severity, decreased over time, and was not associated with decline in lung function [14-16,19]. In extensions of several Phase 2 studies, patients appear to be satisfied with INH, as evidenced by more than 85% of subjects choosing to remain on INH [29]. Subjects also demonstrated a preference for INH to SC insulin or OAs when offered the choice [25-28].

This combination of attributes suggests that INH may be a valuable addition to the treatment options available to patients and physicians in attaining glycemic control. However, these data on efficacy and safety cannot address the overall health benefits and pharmacoeconomic impact of INH in society. No randomized trials have examined the potential advantages of increased uptake of insulin due to avoidance of injections.

This paper describes the Real World Trial of INH, which was designed to overcome the limitations associated with traditional study design. The Real World Trial has adopted a novel design in order to estimate the effects of the availability of INH on physician and patient acceptance of insulin, health status (e.g. glycemic control), and health economic parameters.

## 2.0 Methods

### 2.1 Study population

Approximately 700 patients with T2DM, aged 35 to 80 years with poor glycemic control despite treatment with two or more OAs. Abstinence from smoking is required because smoking greatly increases insulin absorption [30,31].

### 2.2 Study design

**Figure 1:**
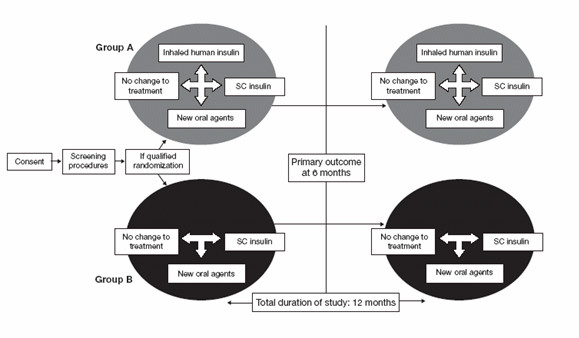
Real World Trial Study Design.

The study is a 52-week, open label, randomized (1:1), parallel, multicenter study consisting of a 6-month core phase to assess change in HbA_1c_, and a 6-month extension phase to assess persistence of effects observed during the initial trial phase. The study design is summarized in Figure 1. Patients will be randomized to one of two experimental groups. In Group A, subjects will be given the option to choose from all appropriate treatment options including SC insulin delivered by either syringe or pen, oral agents, and INH treatment. Study subjects randomized to Group B will have the same treatment options except INH will not be available. In both groups, irrespective of the availability of INH, patients may choose to remain on their current treatment or start any other marketed treatment. Patients and physicians are not under any obligation to use INH in Group A. Choices made at the beginning of the study can be revisited and changed midway through the study.

### 2.3 Outcomes

This trial will provide an estimate of the effect of availability of INH in general practice. The primary end point is change from baseline HbA_1c _(%) after 6 months of treatment between groups. Secondary efficacy end points include:

• HbA_1c _at Week 52

• Proportion of patients achieving adequate glycemic control (HbA_1c _≤ 6.5%, ≤ 7.0%)

• Time-to-insulin therapy initiation

• Change from baseline in fasting plasma glucose level

• Incidence and severity of hypoglycemia

• Change from baseline in body weight and body mass index

• Change from baseline in fasting lipid profile

• Proportion of different treatment categories: INH, SC insulin, changed OAs, no treatment change

• Treatment satisfaction

• Health status

Usual laboratory and clinical monitoring of adverse events will assess safety.

### 2.4 Sample size and analysis plan

A minimum sample size of approximately 279 patients per group will be required to provide 90% power to detect a difference between groups in the primary end point HbA_1c _of at least 0.33% assuming that the standard deviation does not exceed 1.2%. To protect against a possible drop-out rate as high as 10%, approximately 650 patients need to be enrolled.

The primary outcome will be analyzed by intention to treat, using a mixed model. The primary analysis will include treatment and number of OAs as fixed effects. It also will include study centers as random effects due to the large number of anticipated sites (approximately 90), and baseline HbA_1c _(defined as the Week -1 HbA_1c_) will be a patient-level covariate. A similar approach will be used for other continuous outcome measures. Analogous nonlinear mixed models will be used to estimate the effect of random allocation on dichotomous outcomes, incorporating study centers as random effects. An unstratified Cox Proportional Hazards model will be used to estimate the effect of random allocation on time-to-insulin initiation.

### 2.5 Study organization

The study has been accepted by regulatory authorities and IRBs/IECs where the study is being conducted without request for change. All laboratory data are handled by a central contract laboratory. Quality assurance is being provided by a global contract research organization. A standing external advisory committee has provided input into the design and conduct of the study since inception of the aforementioned feasibility study.

## 3.0 Discussion

Estimating the potential benefits of INH on improved insulin acceptance and glycemic control requires adaptation of conventional randomized trial designs. The Real World Trial will estimate the effects of insulin acceptance on glycemic control while preserving the benefits of randomization to reduce bias. Its design takes into account the need for internal and external validity that the results are attributable to the intervention (i.e. INH availability) and that they may be applied to general practice. This design is practical because patients and physicians have real life choices that are normally "designed out" and removed in a "classical" clinical trial. In this trial, physicians are able to freely use the new treatment without some of the restrictions of a traditional trial; physicians and patients can interact normally in discussing, designing, and optimizing treatment regimens. The study also will encompass a variety of clinical practices including community specialist and primary care to better reflect the expected post-marketing use of the product. The findings from the Real World Trial will provide important information that can be generalized for clinicians and healthcare policymakers in distinguishing the role of INH by identifying the association between INH availability/acceptability and glycemic control, and establishing the effects on resource use. Recruitment for the Real World Trial concluded in spring 2006 after recruitment of 739 patients, and results are expected in 2007.

A questionnaire-based feasibility study examining the theoretical effects of the availability of inhaled insulin on treatment in 779 patients with T2DM who were not achieving target glycemic control with their current therapy was recently completed [32]. This randomized, controlled trial provided supportive insight into the design of the Real World Trial, as well as evidence that inhaled insulin may lead to improved uptake of insulin. Treatment preferences expressed by subjects of the feasibility study, by number of OAs, are described in Table 1. In each group, 77% of patients had HbA_1c _values greater than 10%, and the majority of patients were receiving treatment with one or more OA along with dietary and lifestyle advice. Patients were three times more likely to choose insulin therapy when inhaled insulin was available (odds ratio [OR]: 4.16; 95% confidence interval [CI]: 2.93 to 5.95, p < 0.0001) and significantly fewer patients in the group offered inhaled insulin chose to make no change to their therapy compared with patients offered standard treatments only (OR: 0.49; 95% CI: 0.36 to 0.67, p < 0.0001). The proportion of patients choosing insulin in both groups increased with the number of OAs currently being taken, and inhaled insulin was the most frequently chosen treatment option. Aversion to incorporating SC insulin into therapy remained strong, despite high HbA_1c _levels, and the availability of insulin pens in some countries. The enhanced willingness of those offered the option of inhaled insulin treatment to change to a more appropriate therapy increases the potential for achieving improved glycemic control and reducing complications, associated morbidity, premature mortality, and increased cost of diabetes.

**Table 1:** Results from the Real World Feasibility Study [32].

**Current treatment**	**Percentage of study patients (%)**		**Treatment preferences (%)**							
			
			"I will NOT change my current treatment"		"I am willing to consider new oral agents"		"I am willing to consider SC insulin"		"I am willing to consider inhaled insulin"	
	
	Inhaled insulin **available**	Inhaled insulin **NOT available**	Inhaled insulin **available**	Inhaled human insulin **NOT available**	Inhaled insulin **available**	Inhaled insulin **NOT available**	Inhaled insulin **available**	Inhaled insulin **NOT available**	Inhaled insulin **available**	Inhaled insulin **NOT available**
Diet/exercise	**5.4**	7.2	**28.6**	39.3	**47.6**	60.7	**4.8**	0	**4.8**	N/A
1 oral agent	**32.5**	36.6	**26.0**	41.5	**33.9**	43.0	**5.5**	14.1	**30.7**	N/A
2 oral agents	**52.4**	46.1	**28.8**	45.3	**19.0**	35.8	**8.3**	17.9	**38.5**	N/A
3 oral agents	**9.7**	10.1	**23.7**	43.6	**5.3**	33.3	**15.3**	20.5	**50.0**	N/A

One of the key decisions that required consideration in the design of the Real World Trial was the question of the appropriateness of patient- or cluster-level randomization. Rather than allocating patients between treatment groups using a random process, cluster-level randomized trials allocate patients in groups [33]. The rationale for cluster-level randomized trials is apparent when treatments cannot be applied to individuals, for example in a trial of fluoridation of water supplies. Similarly, a cluster trial would be appropriate when there is a risk of contamination of treatments between groups, for example where a healthcare professional was expected to provide different levels of healthcare promotion advice to different patients.

Thus, cluster-level randomized trials are necessary in certain circumstances, but they are associated with a certain disadvantage – we cannot consider patients allocated in this way to be independent of each other. This lack of independence must be addressed in the analysis. The degree to which clustering affects the design of a study depends upon the extent to which patients are clustered within their units of allocation and the number of patients per unit. However, patient per patient, a clustered trial always will be less efficient than an individual patient randomized trial. In addition, there are potential problems associated with the implementation of cluster-level randomized trials. For example, when a trial intervention is considered particularly attractive or of interest to investigating clinicians, the disappointment engendered from being allocated to the control rather than experimental condition may affect the behavior of the investigator. Thus, control group investigators may recruit fewer patients or different patients from those recruited by the experimental group investigators, or they may experience greater loss to follow up. The treatment of interest in the Real World Trial may be applied to individual patients without the risk of contamination; at the time of the study enrollment INH was available only as an investigational product. Thus, there is no requirement to move to a cluster design and the associated issues regarding trial quality and loss of efficiency.

## 4.0 Conclusion

The novel design of the Real World Trial, randomizing patients to scenarios where different treatment options are available for the management of their condition, is necessary in order to provide an estimate of the potential utility of the intervention in standard practice. Trials required for the registration of a new product generally do not provide such information, which is increasingly necessary for healthcare policymakers and clinicians in an environment where healthcare resources are limited. It is very likely that we will see many more such trials in the future.

## Competing interests

Nick Freemantle has received funding from Pfizer Inc for his work on the design of this trial.

Thomas Strack is an employee of the sponsor, Pfizer Inc.

## Authors' contributions

NF and TS conceived of the study and participated in its design and helped to draft the manuscript. All authors read and approved the final manuscript.
